# Dissecting the role of RNA modification regulatory proteins in melanoma

**Published:** 2019-06-04

**Authors:** Parmanand Malvi, Biao Wang, Shreni Shah, Romi Gupta

**Affiliations:** ^1^ Department of Pathology, Yale University School of Medicine, New Haven, CT, 06510, USA; ^2^ Department of Biochemistry and Molecular Genetics, University of Alabama at Birmingham, Birmingham, AL, 35233, USA

**Keywords:** RNA modifications, epitranscriptome, melanoma, MAPK, BRAF mutant melanoma

## Abstract

Melanoma is the deadliest form of skin cancer. Despite recent advances in medicine and the development of new treatments for melanoma, cures remain elusive as acquired resistance to both targeted and immunotherapies are becoming common. Therefore, more studies are conducted to dissect underlying molecular mechanisms that drive melanoma growth in order to provide better therapeutic option. Here, employing a comprehensive and unbiased analysis of different RNA modification regulatory proteins using various publicly available databases we identify the most relevant RNA modifying proteins that plays crucial role in melanoma development. Our study started with the analysis of various genetic alterations (amplifications, mutations/deletion) as well as RNA overexpression of these RNA modification regulatory proteins in The Cancer Genome Atlas melanoma database. We then analyzed their expression in The Human Protein Atlas data. The result of analysis revealed that only a subset of RNA modification regulatory proteins are overexpressed in >75% of melanoma patient cases as compared to normal skin. However, when examined in Oncomine dataset we found only two genes (METTL4 and DNMT3A) were significantly overexpressed in melanoma samples versus normal skin samples and matched with the results of The Human Protein Atlas data. Therefore, we functionally validated METTL4 and DNMT3A using shRNA-mediated knockdown and found that their knockdown in melanoma cells led to melanoma cells growth inhibition. Collectively, in this study, we investigated the epitranscriptomic landscape of melanoma using various publicly available database and identified DNMT3A and METTL4 as the most relevant potential regulators of melanoma growth.

## INTRODUCTION

Melanoma is the deadliest form of skin cancer that account for over 80% skin cancer-related death [1]. Once metastasized, melanoma becomes difficult to treat and significantly impact the survival of melanoma patients [2]. Based on the genomic DNA analysis, melanoma are categorized into four major subtypes that include BRAF-mutant, NRAS-mutant, NF1-deleted/mutated or triple negative melanoma [3-7]. Among these, activating mutations in BRAF and NRAS gene and inactivating mutations in NF1 gene accounts for over 80% of melanoma and results in the activation of MAP kinase pathway [8, 9].

Based on the findings that MAPK pathway is activated in a large percentage of melanoma, clinically effective BRAF kinase and MEK1/2 kinase inhibitors have been developed and are being used to treat metastatic melanoma patients with BRAF mutations [10, 11]. Similarly, immunotherapies have shown durable benefits in some melanoma patients [12, 13]. However, acquired resistance to targeted and immunotherapies is frequent and in part contributes to the treatment failure [14-17]. Therefore, even with advances in precision therapy approaches and success of immunotherapies, melanoma remains a difficult disease to treat with less than 10% five-year survival rate for patients with lymph node metastasis and less than 5% five-year survival rate for melanoma patients with distant metastasis [18-22].

Similar to DNA and proteins, different RNAs can also undergo post-transcriptional modifications that affect their stability, localization and/or function [23]. For example, transfer RNAs (tRNAs) have been shown to contain most extensive and diverse types of modification [24-26]) while ribosomal RNAs (rRNAs) and non-coding RNA are also shown to contain many different post-transcriptional modifications [27]. Similar to other RNAs, mRNAs also contain different types of internal modifications such as *N^6^*-methyladenosine (m^6^A), 5-methylcytosine (m^5^C), 5-hydroxymethylcytosine (hm^5^C) and inosine (I). Because these modifications affect the mRNA stability, localization and/or function of various RNA species, deregulations in these processes are implicated in various pathological conditions, including cancer, cardiovascular diseases and neurological disorders [28].

In this study, employing comprehensive analysis of different RNA modification regulatory proteins using various publicly available databases we identify the most relevant RNA modifying proteins that plays crucial role in melanoma development. Our study started with the analysis of various genetic alterations (amplifications, mutations/deletion) as well as RNA overexpression of these RNA modification regulatory proteins in The Cancer Genome Atlas melanoma database. We then analyzed the expression profile of these in RNA modification regulatory proteins in The Human Protein Atlas data. The result of analysis revealed that only a subset of RNA modification regulatory proteins are overexpressed in >75% of melanoma patient cases as compared to normal skin. However, when examined in Oncomine dataset we found only two genes (METTL4 and DNMT3A) were significantly overexpressed in melanoma samples versus normal skin samples and matched with the results of The Human Protein Atlas data. Therefore, we functionally validated METTL4 and DNMT3A using shRNA-mediated knockdown and found that their knockdown in melanoma cells led to melanoma cells growth inhibition. Collectively, in this study, we investigated the epitranscriptomic landscape of melanoma using various publicly available database to identify the most relevant RNA modification regulatory proteins necessary for melanoma growth and validated DNMT3A and METTL4 as potential regulators of melanoma growth.

## RESULTS

### Analysis of TCGA dataset for N1-methyladenosine (m^1^A), N6-methyladenosine (m^6^A) and 5-methylcytosine (m^5^C) RNA modification regulatory proteins in melanoma

**Figure 1:**
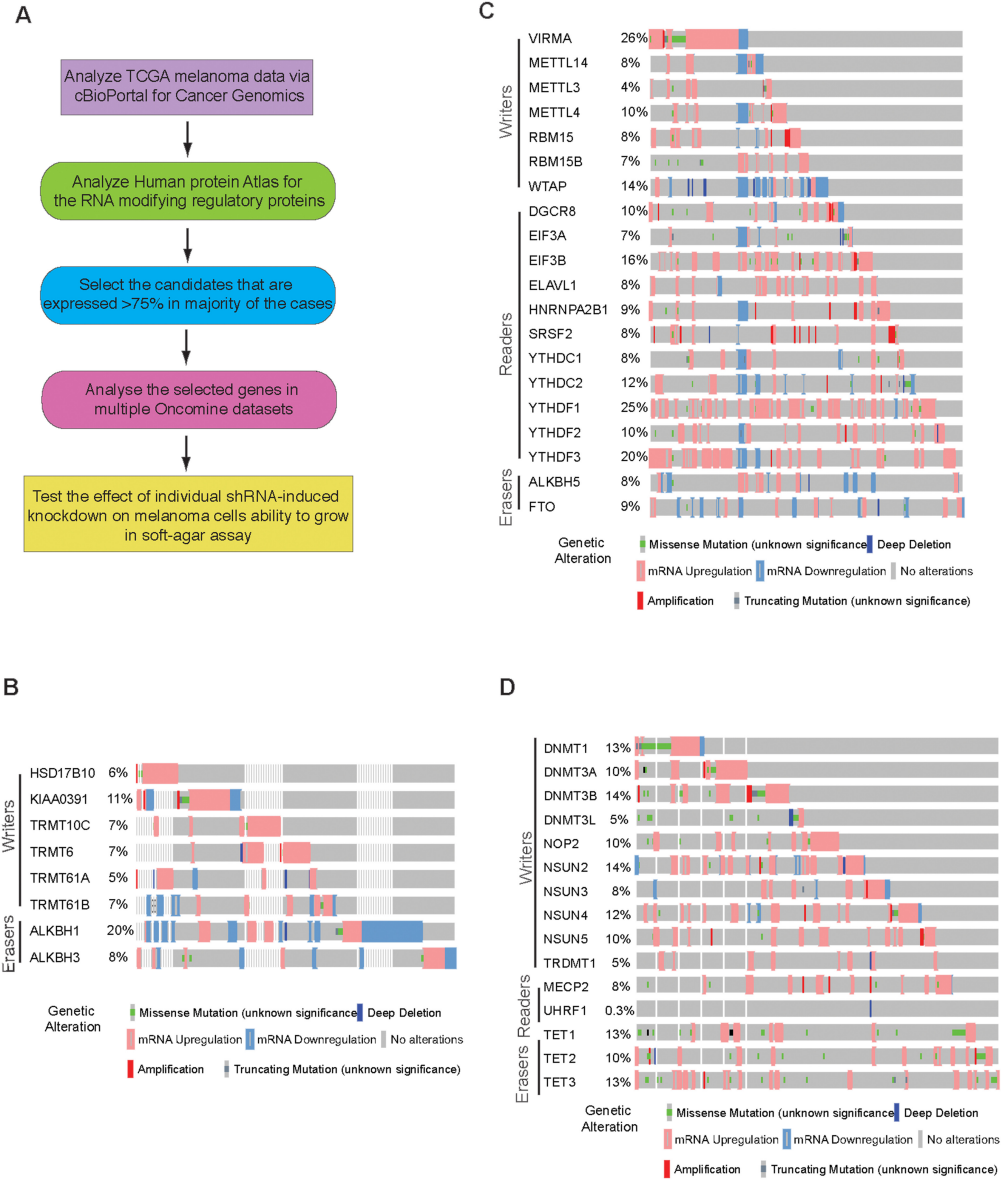
Analysis of m^1^A, m^6^A and m^5^C RNA modification regulatory proteins in melanoma. **(A)** Schematics showing the steps of comprehensive bioinformatic analysis. **(B)** cBioportal analysis of TCGA datasets for the indicated m^1^A RNA modifying genes. **(C)** cBioportal analysis of TCGA datasets for the indicated m^6^A RNA modifying genes. **(D)** cBioportal analysis of TCGA datasets for the indicated m^5^C RNA modifying genes.

Alteration in RNA modification can originate from altered activity or expression of enzymes involved in this process. In order to understand the expression and function of abundant RNA modification regulatory proteins we performed the comprehensive bioinformatics analysis of these using publicly available databases as shown in Figure 1A. Our focus was to analyze RNA modification regulatory proteins that belong to m^1^A, m^6^A, m^5^C, hm^5^C, inosine and other RNA modifying protein group. m^1^A modification regulatory proteins consists of writers and erasers. m^1^A is a reversible modification of tRNA and mRNA. It occurs at positions 9, 14, and 58 of tRNA. mRNA and long-noncoding RNA have been shown to contain hundreds of m^1^A modifications [29, 30]. These modifications are also shown to be present in mitochondrial genes [31-33]. m^1^A modification is a repressive mark in mitochondria and is shown to inhibit the translation [31]. m^1^A writers consist of HSD17B10, KIAA0391, TRMT10C, TRMY6 and TRMT61B; while the erasers include ALKBH1 and ALKBH2.

The expression of m^1^A writers and erasers were analyzed in The Cancer Genome Atlas (TCGA) melanoma data via cBioPortal for Cancer Genomics (TCGA, Provisional; 287 patient samples [34]. The cBioPortal for Cancer Genomics dataset provides visualization and analysis of molecular profiles and clinical attributes from large-scale cancer genomics data sets. This portal shows DNA copy-number data (putative, discrete values per gene, e.g. “deeply deleted” or “amplified”, as well as log2 levels), mRNA and microRNA expression data, non-synonymous mutations, protein-level and phosphoprotein level (RPPA) data, DNA methylation data, and limited de-identified clinical data. Using the TCGA melanoma dataset, we identified various genetic alterations in m^1^A writers and erasers in melanoma (Figure 1B).

N6-methyladenosine modification of RNA (also known as m^6^A) is also the most abundant internal modification of mRNA. In this modification, adenosine base of RNA is methylated at the nitrogen-6 position. m^6^A is a reversible and dynamic modification that regulates key biological processes such as RNA metabolism, embryonic development, and stem cell self-renewal [35, 36]. m^6^A RNA modification regulatory proteins include writers of m^6^A (KIAA1429, METTL14, METTL3, METTL4, RBM15, RBM15B, WTAP), readers of m^6^A (DGCR8, EIF3A, EIF3B, ELAVL1, HNRNPA2B1, HNRNPC1, HNRNPC2, SRSF2, YTHDC1, YTHDC2, YTHDF1, YTHDF2, YTHDF3) and erasers of m^6^A (ALKBH5, FTO).

Previous high-throughput sequencing to identify the sites of m^6^A modifications found that m^6^A modification of RNA was enriched at specific transcript landmarks and were not randomly distributed [37-40]. These studies have shown that m^6^A modifications were typically found clustered around stop codons, in 3’UTRs, and within long internal exons and play a role in translational control [41, 42]. Additionally, m^6^A modifications in mRNAs or non-coding RNAs also play essential roles in various biological processes including embryonic stem cell maintenance and differentiation, meiotic progression, circadian rhythm, heat shock response, and neuronal function [43-45]. When the expression of m^6^A RNA modification regulatory proteins (writers, readers and erasers) was checked in cBioPortal for Cancer Genomics, we found that similar to m^1^A modification regulators, the expression of m^6^A RNA modification regulatory proteins are also widely altered in melanoma (Figure 1C). The alteration level of various m^6^A RNA modification regulatory proteins varied from 4% to 26% in reported melanoma cases.

m^5^C is methylation of RNA has been reported to be abundant in tRNA and rRNA and is shown to stabilizes the RNA secondary structure and affects translational fidelity [46]. Recent advancements have shown them to be present in both mRNA and other lncRNA[47, 48]. It is a dynamic and reversible modification and is catalyzed by the methyltransferases DNMT2 and NSUN2 in human cells. m^5^C modification of RNA occurs at position 5 of cytosine. This have been shown to play role in mRNA export and post-transcriptional regulation [49]. m^5^C RNA modification regulatory proteins consists of writers (DNMT1, DNMT3A, DNMT3B, DNMT3L, NOP2, NSUN2, NSUN3, NSUN4, NSUN5, TRDMT1), readers (MECP2, UHRF1) and erasers (TET1, TET2, TET3). We also tested if writers, reader or erasers of m^5^C were altered in melanoma. TCGA melanoma dataset revealed that a large percentage of m^5^C RNA modification regulatory proteins were upregulated in melanoma, with some also showing mutations, although not recurrent (Figure 1D).

### Analysis of TCGA dataset for 5-hydroxymethylcytosine (hm^5^C), insoine and other RNA modification regulatory proteins in melanoma

**Figure 2:**
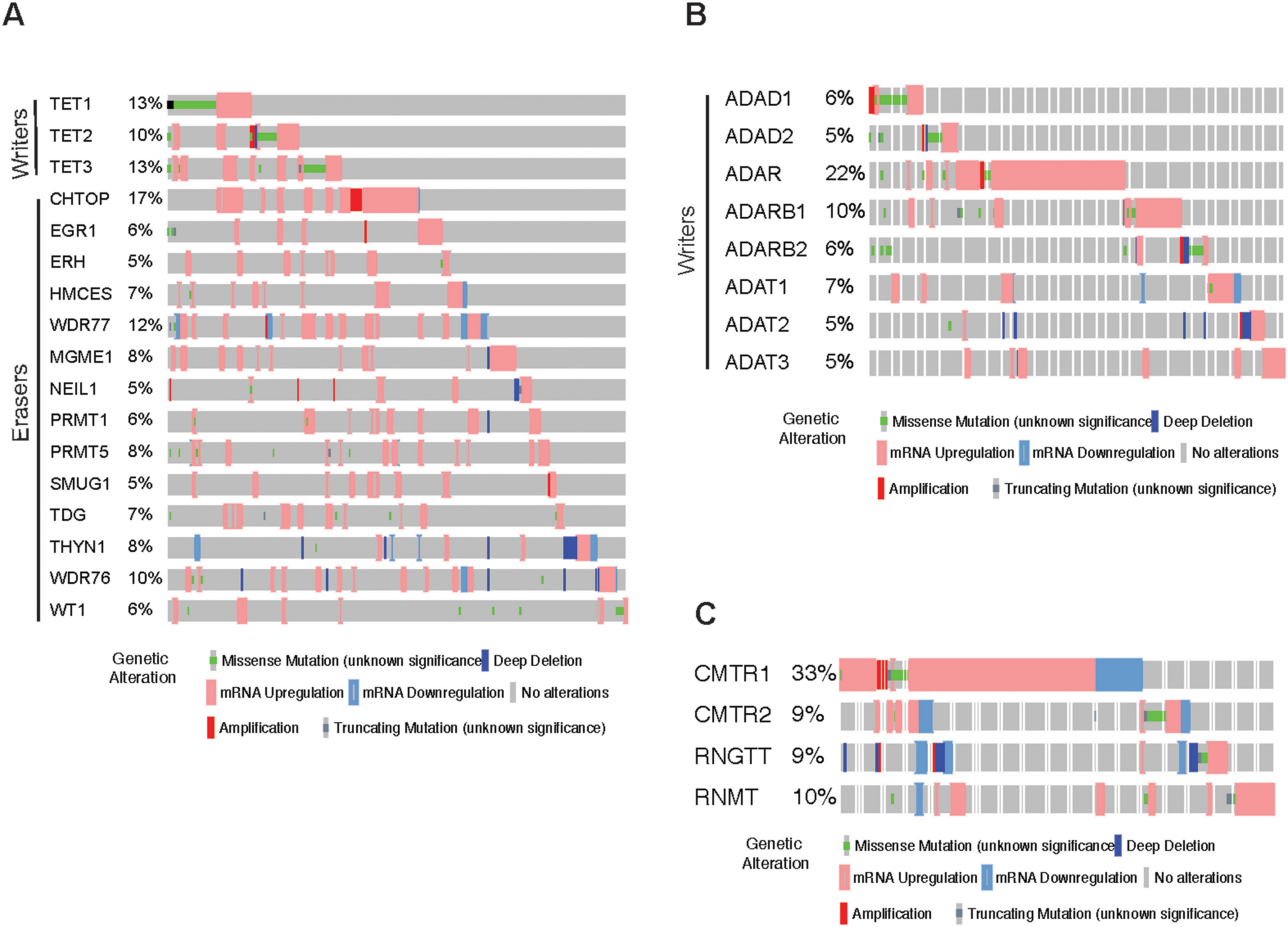
Analysis of hm^5^C, Inosine and other RNA modification regulatory proteins in melanoma. **(A)** cBioportal analysis of TCGA datasets for the indicated hm^5^C RNA modifying genes. **(B)** cBioportal analysis of TCGA datasets for the indicated Inosine RNA modifying genes. **(C)** cBioportal analysis of TCGA datasets for the indicated other RNA modifying genes.

m^5^C in RNA can be oxidized by TET family enzymes to 5-hydroxymethylcytosine (hm^5^C). These modifications are largely associated with polysome enriched RNA and is found to increase translation efficiency [50, 51]. hm^5^C RNA modifying enzyme/proteins group consists of writers (TET1, TET2, TET3) and erasers (CHTOP, EGR1, ERH, HMCES, WDR77, MGME1, NEIL1, PRMT1, PRMT5, SMUG1, TDG, THYN1, WDR76, WT1). We found that the expression of various hm^5^C modifying enzyme/proteins writers, which includes writers and erasers to be altered in multiple melanoma cases (Figure 2A). Some of them such as TET1 and TET3 showed alteration in over 13%-10% of melanoma samples.

Inosine is the one of the most prevalent modification of double-stranded RNA (dsRNA), where adenosine via hydrolytic deamination of the 6-position is converted to inosine and is mediated by ADAR family enzymes. This modification causes the RNA duplex to unwind and also can result in a decrease or an increase in base pairing of the dsRNA substrate depending upon the sequence context [52]. This modification also changes the informational content of the RNA molecule, as inosine preferentially base pairs with cytidine and is therefore interpreted as guanosine by the translational and splicing machinery [53]. And, hence this modification has the double effect. Inosine modification of RNA is carried out by a group of proteins which consist of writers of this mark; ADAD1, ADAD2, ADAR, ADARB1, ADARB2, ADAT1, ADAT2, ADAT3. As a first step, we analyzed the expression for the Inosine RNA modifying regulators using in The Cancer Genome Atlas melanoma database. Among Insoine modifying regulatory proteins strikingly, we found upregulation of ADAR in over 22% samples of melanoma (Figure 2B).

Apart from the known group of RNA modification regulatory proteins, there are some other modifying RNA modification regulatory proteins that are still not categorized. These include CMTR1, CMTR2, RNGTT and RNMT. Similar cBioportal analysis shows that these genes are highly altered in melanoma samples (Figure 2C). In fact, CMTR1 is alerted in 33% of the melanoma cases majority being mRNA upregulation clearly pinpointing that its alteration might play crucial role in this cancer type.

### Analysis of RNA modification regulatory proteins expression in The Human Protein atlas data

**Figure 3:**
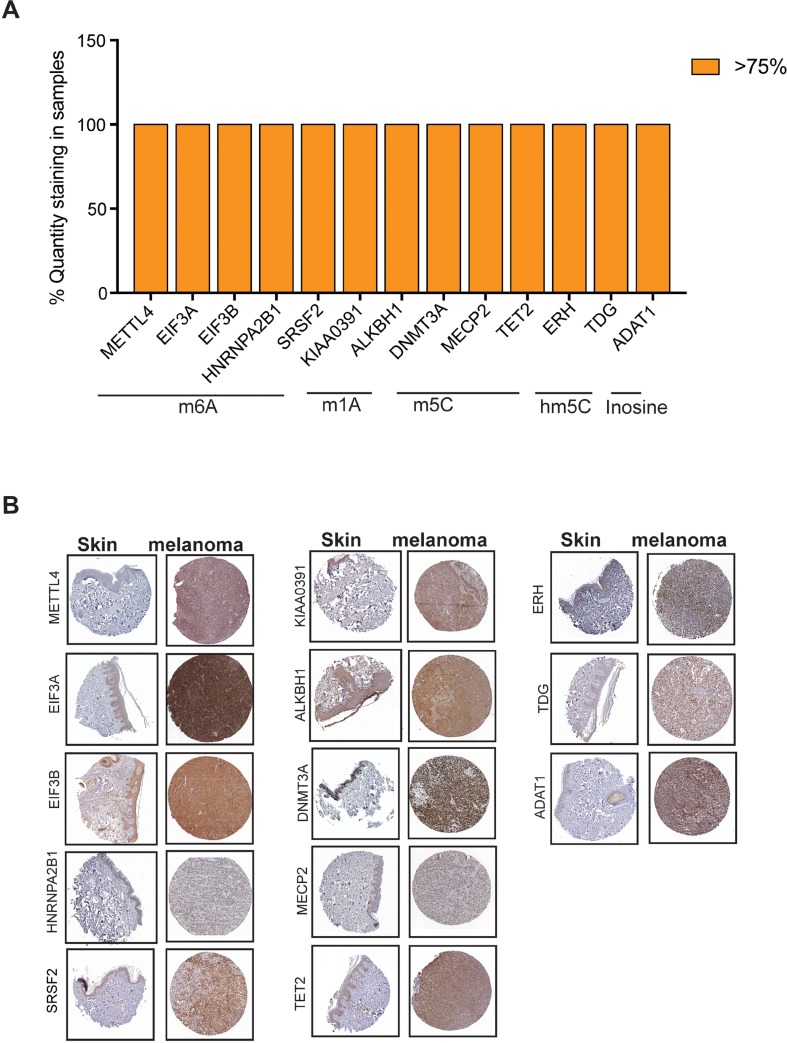
Analysis of expression of RNA modification regulatory proteins in melanoma using The Human Protein Atlas. **(A)** Analysis of protein expression of the indicated m^5^C RNA modifying genes in patient-derived melanoma samples based on immunohistochemical staining intensity using The Human Protein Atlas database. Candidates whose expression was found to be >75% was plotted **(B)** Representative images of immunohistochemical staining of the indicated RNA modifying genes in patient-derived melanoma samples as well as skin from The Human Protein Atlas data.

In order to determine if there is a correlation of various RNA modification regulatory proteins between DNA/RNA levels observed in cBioPortal for Cancer Genomics and their protein levels, we analyzed the protein expression of various RNA modification regulatory proteins in The Human Protein Atlas data. The Human Protein Atlas contains spatial proteomics data for various proteins (immunohistochemistry on tissue microarrays) [54]. Protein profiling using immunohistochemistry allowed us to measure differential protein expression between tumors and corresponding normal tissue counterparts. The results obtained from immunohistochemistry of melanoma tissues from The Human Protein Atlas showed that only small number of RNA modification regulatory proteins are highly expressed (>75%) in majority of the cases (Figure 3A). As shown in Figure 3A, RNA modification regulatory proteins that were expressed in >75% of cases were KIAA0391 and ALKBH1 that are known to regulate m^1^A modification, METTL4, EIF3A, EIF3B, HNRNPA2B1 and SRSF2 that are known to regulate m^6^A modification, DNMT3A, MECP2 and TET2 that are known to regulate m^5^C modification, ERH and TDG that are known to regulate hm^5^C modification and ADAT1 that is known to regulate Inosine modification.

IHC staining for the melanoma samples for these RNA modification regulatory proteins demonstrate that these proteins were significantly overexpressed in melanoma samples as compared to normal skin (Figure 3B). These results indicated that only few RNA modification regulatory proteins whose RNA show upregulation in cBioPortal for Cancer Genomics are also upregulated and correlated with their increased protein levels in melanoma patient samples.

### Analysis of RNA modification regulatory proteins expression in oncomine dataset

**Figure 4:**
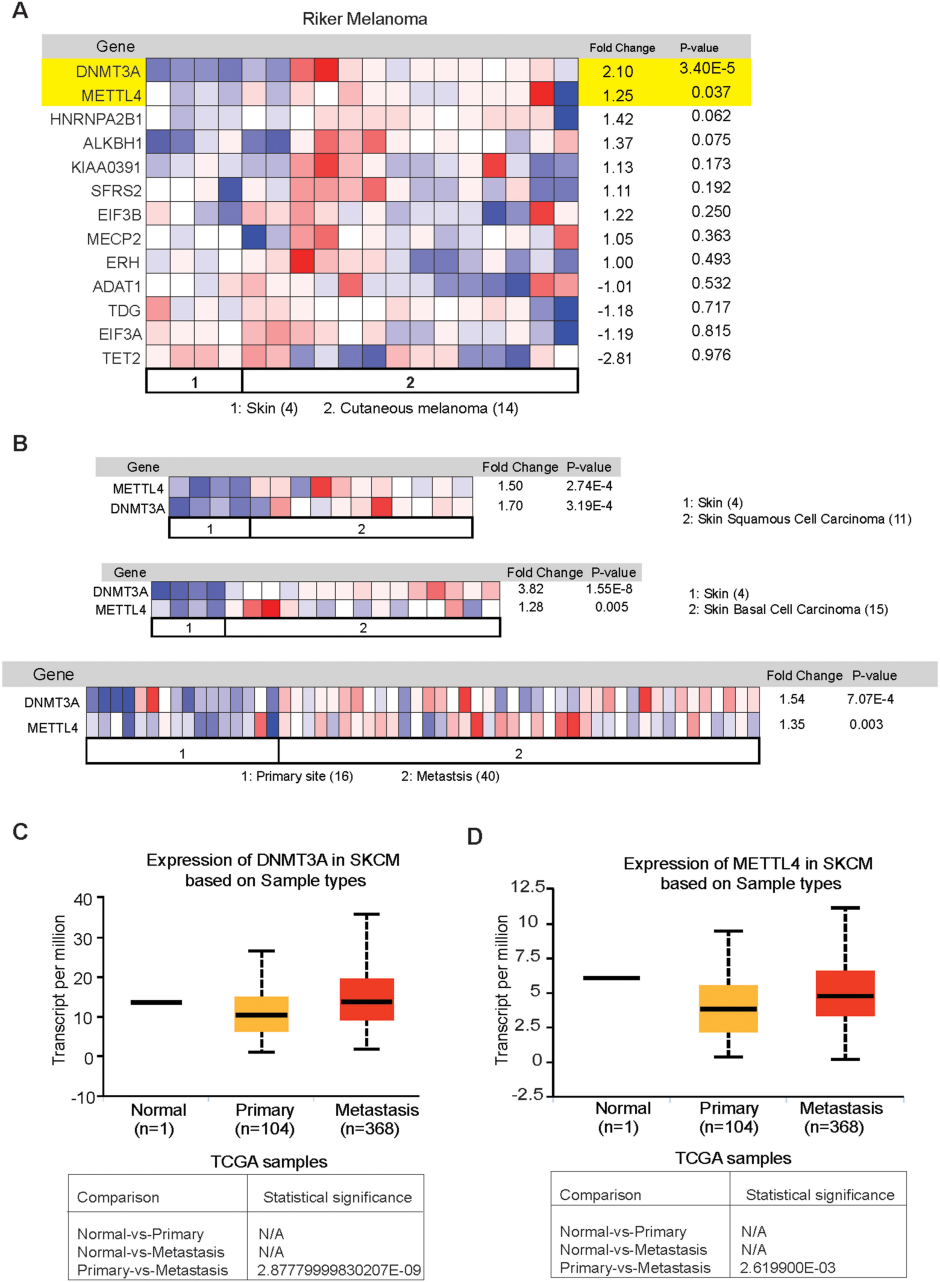
Analysis of expression of RNA modification regulatory proteins in melanoma using The Oncomine dataset. **(A)** The Riker melanoma dataset was analyzed for the expression of the indicated RNA modifying genes in 14 cutaneous melanoma samples and 4 normal skin samples. The relative expression of the indicated RNA modifying genes in patient-derived melanoma samples was compared with normal skin. **(B)** The Riker melanoma dataset was analyzed for DNMT3A and METTL4 expression in 11 skin squamous cell carcinoma, and 15 skin basal cell carcinoma and compared with 4 normal skin samples. DNMT3A and METTL4 expression was also analyzed in 40 metastasis samples and compared with 16 primary site samples. **(C)** Analysis of expression of the indicated DNMT3A RNA modifying genes in UALCAN dataset **(D)** Analysis of expression of the indicated METTL4 RNA modifying genes in UALCAN dataset.

In addition to the TCGA and The Human Protein Atlas data, we also analyzed Oncomine dataset to assess the expression of the proteins that showed overexpression in over 75% of melanoma samples based on The Human Protein Atlas data analysis. Oncomine is a cancer microarray database and web-based data-mining platform [55]. Its goal is to promote gene discovery using genome-wide expression analyses. In this study our aim was to explore the expression of RNA modification regulatory proteins in cutaneous melanoma. In line with that we have chosen Skin cutaneous melanoma (TCGA, provisional) samples for DNA/RNA alteration analysis and in oncomine database we chose “Riker” dataset [56]. It contains cutaneous melanoma, skin squamous cell carcinoma, skin basal cell carcinoma as well as in melanoma metastatic samples. We found that among the set of RNA modification regulatory proteins that are found to be overexpressed in The Human Protein Atlas data, only two candidate genes show significant overexpression in Riker oncomine dataset (Figure 4A) in cutaneous melanoma sample. However, we also observed that both DNMT3A and METTL4 are significantly overexpressed in skin squamous cell carcinoma, skin basal cell carcinoma as well as in melanoma metastatic samples in Riker melanoma dataset set (Figure 4B and Supplementary Figure 1).

Since we observed that DNMT3A and METTL4 were overexpressed in metastatic samples as compared to primary (Figure 4B) we utilized another independent database called UALCAN [57] for similar analysis. UALCAN is an online database for analyzing cancer transcriptome data from TCGA and MET500 cancer transcriptome sequencing data. Analysis of the large scale transcriptome data allow the researchers to identify the most relevant cancer target gene and perform follow up functional validation studies to establish the function of selected genes. UALCAN also provides detailed information on expression of genes in different cancer types, their expression at various stages of cancer, and in cancer subtypes. This dataset also allow the survival analysis in the context of a given gene. We observed that both METTL4 and DNMT3A were significantly upregulated in metastatic melanoma samples as compared to primary tissue (Figure 4C). The results obtained in both Riker and UALCAN datasets show that both DNMT3A and METTL4 expression in upregulated in metastatic melanoma samples and hence these genes may play an important role in melanoma growth and progression.

### METTL4 and DNMT3A are necessary for melanoma growth

**Figure 5:**
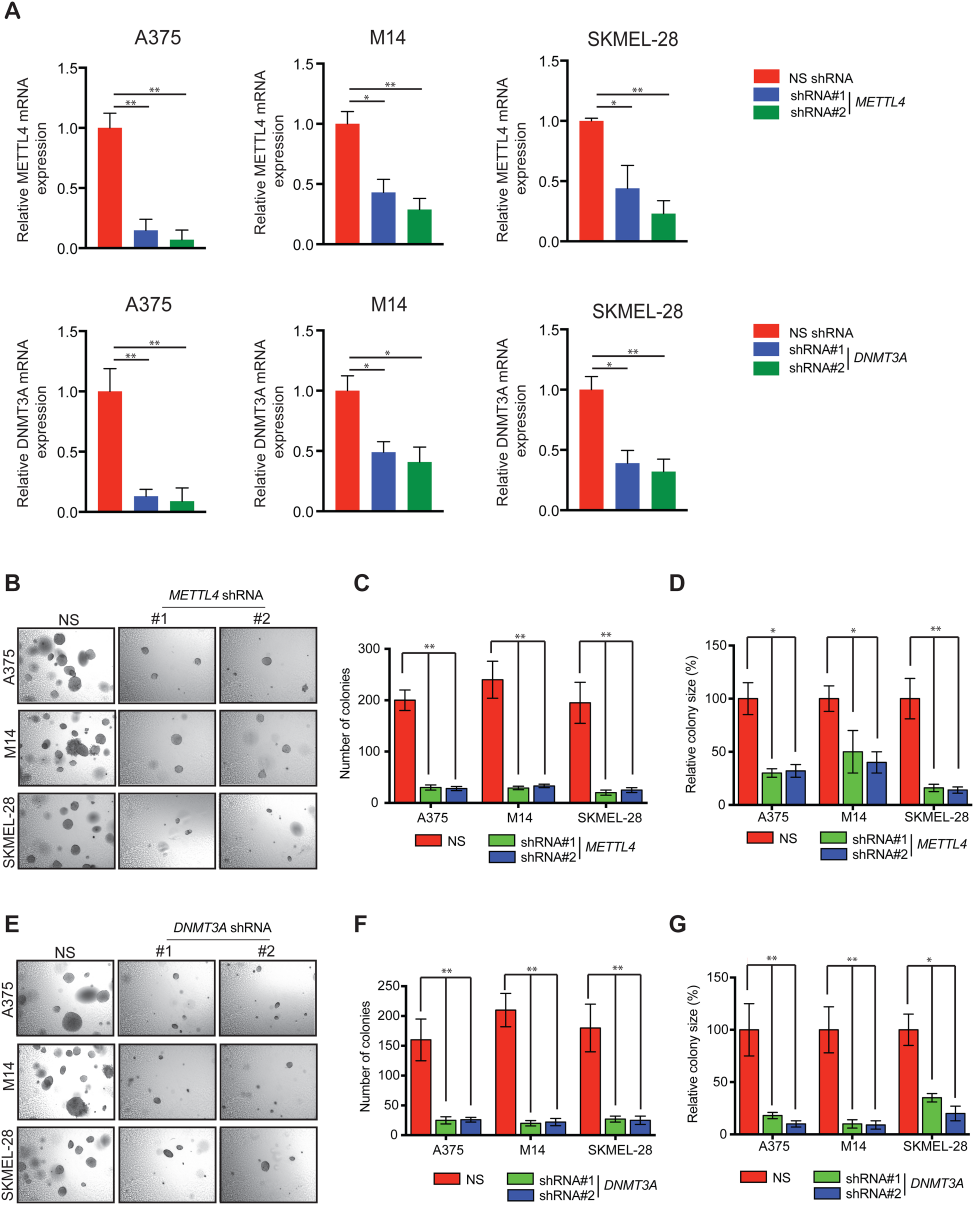
Knockdown of METTL4 and DNMT3A and soft agar growth in melanoma cells. **(A)** A375, M14 and SKMEL-28 cells expressing either METTL4 and DNMT3A or non-specific (NS) shRNA were analyzed by RT-qPCR. Expression of each gene was calculated in the knockdown cells relative to NS shRNA. Actin was used as internal control. Data are presented as mean ± SEM; ^*^, and ^**^ represent p values <0.05and <0.01, respectively. **(B)** The indicated melanoma cell lines expressing METTL4 shRNA were analyzed for colony forming potential using a soft agar assay. Representative images of the soft agar assay under indicated conditions are shown. **(C** and **D)** Relative colony number and size of the indicated melanoma cell lines expressing METTL4 shRNA that were analyzed using a soft agar assay as shown in 5B in A375, M14 and SKMEL-28 cells. **(E)** The indicated melanoma cell lines expressing DNMT3A shRNA were analyzed for colony forming potential using a soft agar assay. Representative images of the soft agar assay under indicated conditions are shown. **(F** and **G)** Relative colony number and size of the indicated melanoma cell lines expressing DNMT3A shRNA that were analyzed using a soft agar assay as shown in 5E in A375, M14 and SKMEL-28 cells.

Our comprehensive analysis on the RNA modification regulatory proteins led us to conclude that METTL4 and DNMT3A are the candidates that showed consistent increase in the RNA as well as protein expression of melanoma samples using TCGA, Oncomine and The Human Protein Atlas data. Next, we asked if these RNA-modifying enzyme/proteins (METTL4 and DNMT3A) that are overexpressed in melanoma samples are necessary for its growth. To this end, we knockdown their expression using shRNA in multiple melanoma cells (Figure 5A). These cells were tested for their ability to form colonies in soft agar. We found that the knockdown of RNA modification regulatory protein encoding genes (*METTL4* and *DNMT3A*) inhibited the ability of melanoma cells to form colonies in soft agar (Figure 5B-5G). Collectively, these results demonstrate that two candidate RNA modification regulatory proteins that were identified to altered using various publicly bioinformatic database in melanoma samples as compared to normal skin when analysed at functional levels also show to play crucial role in regulating melanoma growth.

## DISCUSSION

Melanoma is the deadliest form of skin cancer, accounting for the majority of the skin-cancer related deaths. Several new therapies [10, 11] including immunotherapies [12, 13] have been developed to target melanoma all with limited success due to the emergence of drug resistance [14-17]. As the number of potential therapeutic DNA targets dwindle, many researchers are turning to RNA to tackle the problem. In recent years, numerous new mRNA modifications have been discovered providing evidence for the existence of an “Epitranscriptome” that might be just as important as the epigenome. RNA modifications occur in mRNA, rRNA and tRNA and other non-coding RNAs affecting wide variety of functions ranging from improving RNA stability to increasing protein diversity to suppressing translation termination. Based on the nature of its functions and the fact that they regulate several important cellular processes. Many studies have shown that these RNA modifications play crucial role in melanoma growth and metastasis [58, 59]. They are also involved in drug resistance mechanism. These studies not only allow us to understand novel pathways that cause melanoma to become untreatable but also pave the way to develop new, effective and sustainable therapeutic tools for optimal drug selection and treatment.

**Figure 6:**
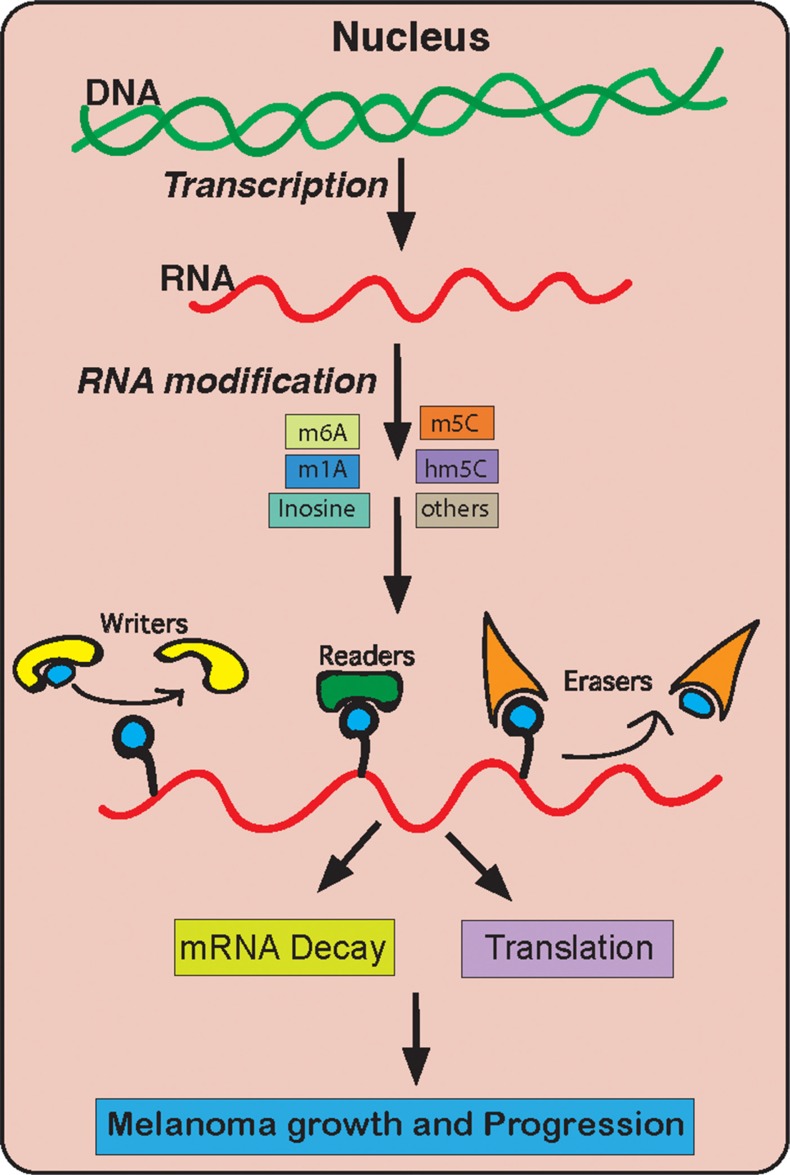
A model summarizing the role of RNA modification genes in melanoma growth and development.

As shown in Figure 6, we focused our studies on different RNA modifying enzyme/proteins. Our goal was to perform a comprehensive study on the expression of these different RNA modification regulatory proteins. These detailed analyses allowed us to understand which of these different RNA modification regulatory proteins are important for melanoma. We used several bioinformatics platforms that are publicly available and contain large amount of information on melanoma patient samples. These databases include information on the DNA, mRNA and their protein in melanoma samples as compared to normal skin. Our initial results showed that many of the genes are upregulated in melanoma according to all of the datasets. Although, the traditional view has been that a driver oncogenic event typically occur as an activating mutation in protooncogene, such as in NRAS and BRAF genes in case of melanoma. However, there is ample evidence that genes that are amplified or overexpressed are also of great importance for cancer growth and progression. For example, Melanocyte Inducing Transcription Factor (MITF) is either overexpressed or amplified in about 13% of cases and is shown to be important for melanoma progression and is a lineage survival oncogene in malignant melanoma [60]. In addition to MITF, other amplified and overexpressed genes are HER2 in 15%–20% of invasive breast carcinomas [61], MYC in 15-20% of breast cancer [62] and several hematological malignancies [63], EGFR in squamous cell carcinomas [64], MDM2 in several cancer type [65]. Based on this we infer that the upregulated expression of RNA modification regulatory proteins METTL4 and DNMT3A play a key role in melanoma initiation or progression. However, much stringent analysis of these candidate genes needs to be performed using multiple platforms that analyses both RNA and protein levels in patient samples. Further, these identified candidate genes also need to be validated using secondary functional assay to illuminate their role in other aspects of melanoma tumor growth and progression (e.g., metastasis). In our study we found out abundant RNA modification regulatory proteins that are overexpressed in melanoma but only a few of them are necessary for melanoma growth.

Taken together, our studies provide a comprehensive signature for alterations in RNA modification regulatory proteins in melanoma. We also performed functional validation studies for DNMT3A and METTL4 and found that they are potentially important for melanoma growth.

Additional future studies are needed to fully determine the role of these RNA modification regulatory proteins in melanoma tumor growth and progression (e.g., metastasis). Since RNA is a key molecule that drives every cellular process, their deregulation is present in nearly all human disease and play a causative role. Therefore, new discoveries that would allow to identify RNA modification regulatory pathways will open up new avenues for cancer treatment.

## MATERIALS AND METHODS

### Cell culture

The cell lines A375, M14, and SKMEL-28 were purchased from the American Type Culture Collection (ATCC) and were grown in Dulbecco's Modified Eagle Medium (DMEM) or RPMI 1640 medium supplemented with 10% FBS and 1% Penicillin/Streptomycin antibiotics.

### shRNAs, transfection, lentivirus preparation, and stable cell line generation

All shRNAs were obtained from OpenBiosystems and are listed in Table 1. Lentivirus particles carrying shRNA were generated by co-transfecting shRNA plasmids with the lentiviral packaging plasmids pSPAX2 and pMD2.G into 293T cells using Effectene (Qiagen) according to the manufacturer’s instructions. Viruses were filtered using a 0.45 μm filter. Stable cell lines were generated by infecting various melanoma cell lines with shRNA lentivirus in 12-well plates followed by puromycin selection (0.2–1.5 μg/ml).

**Table 1:** Primer sequences for RT-qPCR analysis; antibodies used; source and concentration of chemical inhibitors used.

Application	Gene symbol	Forward primer (5¢-3¢)	Reverse primer (5¢-3¢)
	METTL4	ggaagacctgtgggcagctt	aaaatgccccttccagtcca
	DNMT3A	ctacgcaccacctccaccag	caatgttccggcacttctgc
	ACTINB	gcatggagtcctgtggcatc	ttctgcatcctgtcggcaat
**shRNAs**	**Gene symbol**	**Clone ID**	**Catalog number**
	METTL4	TRCN0000034828	RHS3979-9602236
	METTL4	TRCN0000034830	RHS3979-9602238
	DNMT3A	TRCN0000035755	RHS3979-9603163
	DNMT3A	TRCN0000035758	RHS3979-9603166

### RNA isolation, reverse transcription quantitative PCR (RT-qPCR)

For mRNA expression analyses, total RNA was extracted using TRIzol (Invitrogen) and purified using RNeasy Mini Columns (Qiagen), and cDNA was generated using ProtoScript first strand cDNA synthesis kit (New England Biolabs) according to manufacturer’s instructions. Quantitative PCR was then performed using the Power SYBR Green (Master Mix) (Life Technologies). The oligonucleotide sequences used for RT-qPCR are provided in Table 1.

### TCGA from cBioportal analysis datasets analysis

The cBioPortal for Cancer Genomics Web site, http://www.cbioportal.org, was used to access TCGA melanoma patient sample data. Skin Cutaneous melanoma (TCGA provisional) was used for analysis. It contains data from 287 melanoma patient samples. Mutations, copy-number alterations from GISTIC and mRNA Expression z-Scores (RNA Seq V2 RSEM) for each indicated RNA modification regulatory gene was analyzed. Oncoprint of each gene is shown which reflects the genetic alterations such as Amplification, deep deletion, mRNA upregulation, mRNA downregulation, Missense mutation, Truncation mutation, In-frame mutation and also if there is no alteration.

### Oncomine dataset analysis for the mRNA expression of RNA modification regulatory genes

Riker melanoma dataset was downloaded from Oncomine (https://www.oncomine.org), analyzed for RNA expression of mRNA modifying genes. The Riker melanoma dataset analyzed included 4 normal skin samples, 14 cutaneous melanoma samples, 11 skin squamous cell carcinoma samples, 15 skin basal cell carcinoma samples, 16 primary site samples, 40 metastasis samples using an Affymetrix HG U133 Plus 2.0 microarray. Relative expression and their significance are shown in the images download.

### UALCAN analysis of RNA modification regulatory genes:

Expression of RNA modification regulatory genes was downloaded from UALCAN dataset. UALCAN is publicly available at http://ualcan.path.uab.edu. Gene expression is plotted for normal, primary and metastatic samples for skin cutaneous melanoma.

### The Human Protein Atlas data

The Human Protein Atlas [55] is a publicly available data with millions of high-resolution images showing the spatial distribution of proteins detected by 15,598 different antibodies (release 9.0, November 2011) in 46 different normal human tissue types and 20 different cancer types, as well as 47 different human cell lines. Specimens containing normal and cancer tissue have been collected and sampled from anonymized paraffin embedded material of surgical specimens, in accordance with approval from the local ethics committee. The images represent a view similar to what is seen in a microscope when examining sections of tissue on glass slides. Each antibody in the database has been used for IHC staining of both normal and cancer tissue. TMA images of normal and melanoma tissue were downloaded from the HPA (http://www.proteinatlas.org/). Each image is 300 × 300 pixels and represents a section of a tissue core composite of two stains; DAB and hematoxylin. The blue channel corresponds to regions stained with Hematoxylin and is expressed mainly in the nuclei. In that image, most nuclei are also positive and are, therefore, also stained with the brown DAB stain. We also plotted quantity of samples stained in melanoma cases with the specific antibody. The Antibody ID used for IHC staining the samples are provided in Table 2.

**Table 2:** Genes were analyzed for their expression in The Human Protein ATLAS data. Antibody used for IHC staining is listed below

GENES	Antibody id
KIAA0391	HPA020459
ALKBH1	HPA044087
METTL4	HPA040061
EIF3A	HPA038315
EIF3B	HPA048983
HNRNPA2B1	HPA001666
SRSF2	HPA049905
DNMT3A	HPA026588
MECP2	HPA000593
TET2	HPA043135
ERH	HPA002567
TDG	HPA052263
ADAT1	HPA040713

### Soft agar assay

Soft-agar assays were performed by seeding 5 × 10^3^ to 2 X10^4^ melanoma cells stably expressing the indicated shRNA onto 0.4% low-melting-point agarose (Sigma-Aldrich) layered on top of 0.8% agarose. After 3–4 weeks of incubation, colonies were stained with a 0.005% crystal violet solution and imaged using a microscope. Representative soft agar images are shown for each condition. Colony number and colony size was measured using ImageJ software.

### Statistical analysis

All experiments were conducted in at least as three biological replicates. Results for individual experiments were expressed as mean ± SEM. P-values were calculated by t-tests using GraphPad Prism version 7.0a for Macintosh OS X, GraphPad Software, San Diego, California USA (www.graphpad.com). A p-value of less than 0.05 was considered statistically significant (^*^ = p < 0.05, ^**^ = p < 0.01, ^***^ = p < 0.001, ^****^ = p < 0.0001).

## SUPPLEMENTARY MATERIALS TABLE


