# Supplementary material for: Dissecting the role of RNA modification regulatory proteins in melanoma
Source: Oncotarget. 2019 Jun 4;10(38):3745–59. (PMC6557201)
Supplement: Supplementary file 1 [file oncotarget-10-3745-s001.pdf]

Dissecting the role of RNA modification regulatory proteins in melanoma

SUPPLEMENTARY MATERIALS

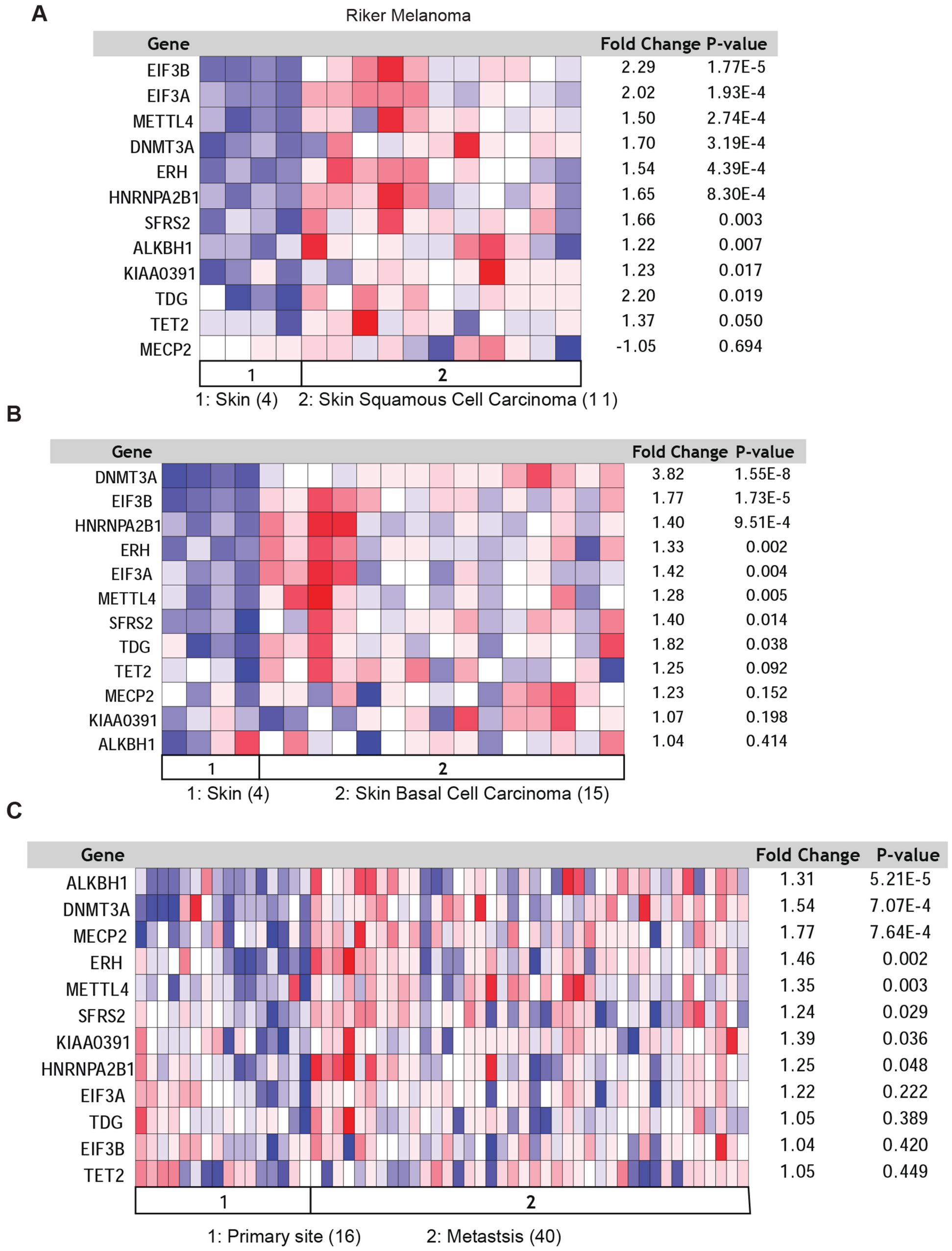

Supplementary Figure 1: Analysis of expression of RNA modification regulatory proteins in melanoma using The Oncomine dataset. **(A)** The Riker melanoma dataset was analyzed for the expression of the indicated RNA modifying genes in 11 skin squamous cell carcinoma and 4 normal skin samples. The relative expression of the indicated RNA modifying genes in patient-derived melanoma samples was compared with normal skin. **(B)** The Riker melanoma dataset was analyzed for the expression of the indicated RNA modifying genes in 15 skin basal cell carcinoma and 4 normal skin samples. The relative expression of the indicated RNA modifying genes in patient-derived melanoma samples was compared with normal skin. **(C)** The Riker melanoma dataset was analyzed for the expression of the indicated RNA modifying genes in 40 metastasis samples and 16 primary site samples.. The relative expression of the indicated RNA modifying genes in metastasis melanoma samples was compared with primary samples.
